# Prenatal exposure to metal mixtures and the risk of overweight and obesity in school-aged children: insights from metabolomic profiling

**DOI:** 10.3389/fnut.2026.1762885

**Published:** 2026-02-24

**Authors:** Xu Yang, Xian Sun, Chun Yan, Wen Zhang, Lili Bao, YuWen Lv, Quanquan Guan, Yanyan Yu, Yankai Xia

**Affiliations:** 1 Department of Pediatrics, Suzhou Affiliated Hospital of Nanjing Medical University, Suzhou Municipal Hospital, Gusu School, Nanjing Medical University, Suzhou, China; 2 Preventive Health Care Department, Ligang Hospital of Jiangyin City, Wuxi, China; 3 State Key Laboratory of Reproductive Medicine and Offspring Health, School of Public Health, Nanjing Medical University, Nanjing, China; 4 Key Laboratory of Modern Toxicology of Ministry of Education, School of Public Health, Nanjing Medical University, Nanjing, China

**Keywords:** children, metabolomics, metal mixture, overweight and obesity, pregnancy

## Abstract

**Background:**

Prenatal metal mixtures exposure was associated with child growth. However, long-term impact of maternal metal mixture exposure on offspring's overweight or obesity (OWO) in childhood and the potential role of metabolites remain poorly understood.

**Methods:**

Based on a prospective cohort study, ten metals were measured and metabolomics profiling was conducted in maternal serum during pregnancy. Children's anthropometric parameters were measured at school age and OWO was defined according to the international World Health Organization (WHO) reference data. A combination of multiple regression models, variable selection models and exposome models were conducted to explore the effects of prenatal metal mixture exposure on child OWO and BMI *z*-score. A meet-in-the-middle (MITM) approach was employed to examine metabolites' potential role in mediating this association.

**Results:**

Maternal metals exposure such as Cu and V was found to be positively associated with OWO risk based on single-metal models, with ORs being 24.171 (95% *CI*: 2.351–403.256) and 2.534 (95% *CI*: 1.273–5.623), respectively. Similarly, prenatal exposure to V was also positively associated with BMI *z*-scores in school-aged children (β: 0.293, 95% *CI*: 0.015–0.572). Marginal association was found between Cu exposure and BMI *z*-scores (β: 0.758, 95% *CI*: −0.001–1.517). Metabolites such as glycerophosphocholine and glycine played potential intermediate roles in the association between maternal Cu levels and OWO risk.

**Conclusion:**

Prenatal Cu and V exposure may have an adverse effect on school-aged childhood's OWO risk, and metabolites may play an important intermediate role. By further examining metabolite profiles, our findings offer insight into potential metabolic pathways through which prenatal metal exposure may influence childhood obesity risk, thereby extending existing epidemiological evidence with a mechanistic perspective. Multi-center population studies and *in vivo* studies in future are needed to validate the results.

## Graphical Abstract

**Figure:**
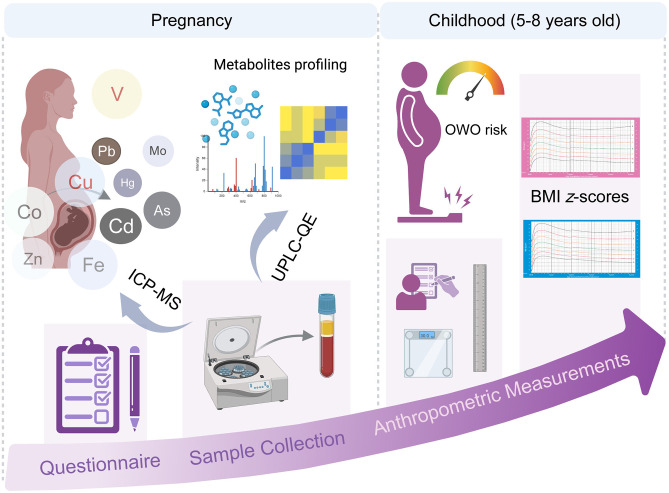

## Highlights

- The associations of prenatal metal exposure, metabolites and pediatric overweight or obesity (OWO) risk were evaluated in a prospective cohort study.
- Metal mixture during pregnancy was associated with an increased risk of OWO, with Cu and V identified as the primary contributor to this joint effect.
- Metabolites such as glycerophosphocholine and glycine were found to be crucial mediators in the association between maternal Cu levels and OWO risk, highlighting potential biological mechanisms.

## 1 Introduction

Pediatric overweight or obesity (OWO) has been an emerging worldwide public health concern, with prevalence rising rapidly over the past few decades. By 2030, an estimated 254 million children are expected to be affected by obesity (1). This condition often persists into adulthood, contributing to long-term health burdens and increasing healthcare costs for individuals and society (2). Although traditional risk factors such as genetic predisposition, excessive caloric intake, and physical inactivity are well recognized (3), growing evidence suggested that the origins of this epidemic may begin at early developmental stages (4). Prenatal and early-life exposure to environmental chemicals, known as “obesogens”, can disrupt metabolic programming during fetal development (5), thereby perpetuating a cycle of obesity across generations (6).

Metals, a class of endocrine-disrupting chemicals, have been shown to exhibit estrogenic activity that may promote fat accumulation (7). Pregnant women may be exposed to metals through common environmental sources such as diet, drinking water, ambient air pollution, and environmental contamination (8). Previous studies have reported measurable concentrations of multiple metals in maternal blood or serum samples in Chinese populations and globally (9, 10). Heavy metals such as lead (Pb) and mercury (Hg) can readily cross the placental barrier, exposing the developing fetus during critical stages of development (11). Growing evidence revealed that prenatal exposure to these metals may influence children's growth and development and increase obesity risk (12–14). A large population-based study found that higher copper (Cu) levels in maternal serum were associated with an increased risk of childhood OWO (15). However, existing findings remain inconsistent across study designs and geographic regions, and most of them have examined only a single category of metals. There is an urgent need to investigate the combined effects of both essential and non-essential metals using a well-designed prospective cohort study.

The biological plausibility of prenatal metal exposure influencing pediatric obesity risk may lie in metal-induced oxidative stress, which has been demonstrated to be associated with insulin resistance and metabolic dysregulation (16). Advances in metabolomics have yielded important insights into the biological pathways linking prenatal metal exposure to subsequent health outcomes (17, 18). One study demonstrated that maternal metal exposure such as Pb, Hg, and Cadmium (Cd) may disrupt fetal metabolic programming (19). Significant perturbation in the maternal metabolome during pregnancy may contribute to an increased risk of childhood obesity (20). A birth cohort study further suggested that disturbances in metabolites found in cord blood could serve as important mediators in the association between vanadium (V) exposure and birth size (21). However, the potential role of prenatal metabolomic profiles as mediators linking prenatal metal mixtures exposure to childhood overweight has not yet been quantified.

Based on a well-established prospective birth cohort study, we measured the levels of ten metals and metabolomic profiles in maternal serum samples, and assessed the growth status of children aged 5–8 years. This study aimed to investigate the effects of prenatal exposure to metal mixtures on OWO and BMI *z*-scores in school-aged children, as well as to elucidate the underlying metabolic perturbations, thereby providing mechanistic insights into this association.

## 2 Materials and methods

### 2.1 . Study population

The study was established at the affiliated hospitals of Nanjing Medical University and details about this population have been previously reported (22). Pregnant women who met the following criteria were recruited: (1) Residents of Nanjing City; (2) Age ≥ 18 years old; (3) Delivering at the study hospital; and (4) No serious disease such as cancer, hepatitis B or psychiatric illness. Initially, a total of 698 pregnant women were recruited in this study and maternal blood samples were collected. Follow-up was conducted when children were aged 5–8 years. Participants were excluded if they (1) had no birth information (*n* = 169); (2) had twins (*n* = 5); (3) were conceived through assisted reproduction technology (*n* = 25); (4) had no anthropometric measures information of children (*n* = 316); (5) had insufficient serum samples (*n* = 53). Finally, a total of 130 mother-child pairs were retained for the final analysis. Ethical approval was obtained from the Institutional Review Board of Nanjing Medical University, and all participants provided written informed consent prior to participation.

### 2.2 Serum collection and metal levels measurement

At enrollment, peripheral blood samples were collected from pregnant participants. Serum was isolated by centrifugation at 3,000 revolutions per minute for 5 min at 4 °C, and subsequently aliquoted and preserved at −80 °C until analysis. Metal levels were quantitatively measured in the collected samples. Quantification of metal concentrations in serum was conducted, with 15 elements initially detected. Among all measured metals, those with acceptable quality control performance and detection rates above 20% were retained for subsequent analyses, consistent with common practice in environmental epidemiology to exclude analytes with low detection frequencies to ensure statistical reliability (23). Of these, ten metals, namely iron (Fe), Cu, molybdenum (Mo), V, zinc (Zn), cobalt (Co), arsenic (As), Hg, Pb and cadmium (Cd), were selected for inclusion in the current study. Analytical procedures adhered to a previously validated protocol (24). Briefly, each serum sample underwent acid digestion using a mixture comprising 0.05% (v/v) Triton X-100, 0.1% (v/v) nitric acid, and internal standards (10 mg/L each of terbium [Tb], indium [In], bismuth [Bi], yttrium [Y], and scandium [Sc]). Metal quantification was performed using an inductively coupled plasma mass spectrometer (iCAP Q, Thermo Scientific, Bremen, Germany). Quality assurance was maintained by incorporating spiked quality control samples—constituting 10% of the sample batch—and procedural blanks, with one blank processed per ten samples. The relative standard deviations (RSDs) for both pooled and quality control samples ranged from 2.1% to 11.1%. Limits of detection (LODs) were estimated as three times the standard deviation derived from ten replicate blank digests. Detection rates of metals were calculated as the percentage of samples in which the measured concentration of a given metal exceeded its respective LOD.

### 2.3 High-resolution metabolomic analysis of maternal serum samples

Metabolomic profiling was conducted following a validated protocol described before **(author?)** (25). In brief, 20 μl of serum was aliquoted into a 1.5 ml Eppendorf tube and extracted with methanol at a 1:3 volume ratio. After vortex mixing for 10 s, and the samples were centrifuged at 15,000*g* for 15 min at 4 °C to precipitate proteins. The collected supernatant was dried under a gentle nitrogen stream and reconstituted in 10 μl of solvent prior to instrumental determination. Metabolite detection was carried out using an Ultimate 3,000 UPLC system (Dionex) coupled with a Q-Exactive mass spectrometer (Thermo Fisher Scientific, Bremen, Germany). Data acquisition was conducted in full-scan mode (resolution 70,000; m/z 70–1,050). Chromatographic separation used a gradient elution with mobile phases of 0.1% formic acid in water (A) and in acetonitrile (B). The flow rate was 0.4 ml/min, and the total run time was 15 min. To minimize analytical bias, sample injections were performed in random order. Metabolites were identified by comparing accurate mass, retention time, and tandem MS (MS/MS) spectra with those in a validated in-house reference library of authentic standards.

### 2.4 Outcomes and covariates definition

At the school-aged assessment (5–8 years), anthropometric measurements, including height and weight, were obtained using standardized and routinely calibrated equipment. Children's age-and-sex-adjusted BMI *z*-scores were derived based on the international World Health Organization (WHO) reference curves (26, 27). According to the WHO growth reference, overweight and obesity in children were defined as age- and sex-adjusted BMI *z*-scores ≥1 and ≥2, respectively (27). For the primary analyses, child OWO was defined as having a BMI *z*-score ≥1, encompassing both overweight and obesity.

Demographic information for both mothers and children were extracted from hospital maternity records by qualified medical personnel. At enrollment, trained interviewers administered standardized questionnaires to obtain sociodemographic and health-related data. Maternal variables included pre-pregnancy BMI, educational attainment, and passive smoking history. Child-related data included age, sex, birth weight, duration of outdoor activity, and frequency of sugar-sweetened beverage intake.

### 2.5 Statistical analysis

Differences between children with OWO and healthy controls were evaluated using independent-sample *t*-tests for continuous variables and Chi-square tests for categorical variables. To normalize the right-skewed distribution of serum metal concentrations, a natural logarithmic transformation was applied before statistical analysis.

Associations between individual metal exposures and the risk of OWO and BMI *z*-scores were examined using multivariable logistic regression models and generalized linear regression models, separately. Potential confounders were selected based on prior evidence and empirical associations with the outcomes (*p* < 0.20). These variables included continuous variables (maternal age, pre-pregnancy BMI, children's birth weight) and categorial variables (history of passive-smoking, maternal education, parity, children's gender, outdoor activity time, and the frequency of sugar-sweetened beverage intake). Metals levels blow the LOD were imputed by LOD/$\sqrt{2}$ (28). Dose-response relationships were further assessed using restricted cubic spline (RCS) functions.

To explore associations between prenatal exposure to metal mixtures and OWO as well as BMI *z*-scores, we applied elastic net (ENET) regression, with model optimization achieved through 10-fold cross-validation (29). Furthermore, the Deletion-Substitution-Addition (DSA) model (30) was also employed, with 50 iterations performed, to identify metals jointly associated with childhood health outcomes. For both models, co-exposure models were then constructed by incorporating all measured metals into a logistic regression framework for OWO and a generalized linear regression model for BMI *z*-scores, adjusting for relevant covariates.

Non-linear and interactive associations among metal mixtures were investigated using Bayesian kernel machine regression (BKMR) model (31). The joint effect of all ten metals was estimated by comparing the OWO risk at the 25th−75th percentile relative to median exposure levels. Each metal's independent effects were evaluated by varying the concentration of one metal while holding all others fixed at their medians. BKMR models were fit using a binomial distribution for OWO and a Gaussian distribution for BMI *z*-scores with 100 knots and 5,000 iterations of Markov Chain Monte Carlo (MCMC) sampling. Metals with correlation coefficients greater than 0.3 were grouped into the same category. Based on Spearman's correlation coefficients, Fe, Zn, and Mo were classified into Group 1; Cu and Co into Group 2; V, Cd, and Pb into Group 4; and Hg into Group 5. Group posterior inclusion probabilities (groupPIP) derived from the BKMR model were used to characterize the overall importance of each exposure group. Conditional posterior inclusion probabilities (condPIP) were calculated to evaluate the relative contribution of individual metals to the OWO risk and BMI *z*-score variation. PIP values greater than 0.5 were considered indicative of a substantial contribution to OWO risk and BMI *z*-score variation. Bivariate exposure–response functions were additionally estimated within the BKMR framework to assess potential interactions between metals, with all remaining metals fixed at their median concentrations.

Associations of metabolites and metal exposures with OWO and BMI *z-*scores were analyzed using generalized linear regression models. To explore potential biological mediation pathways, the meet-in-the-middle (MITM) framework was applied to assess the extent to which metabolites mediated the relationship between prenatal metals exposure and OWO and BMI *z*-scores (32).

In addition, to explore potential sex-specific and age-related differences in the associations between maternal metal exposure and child growth, we conducted stratified analyses by child sex (boys vs. girls) and by age group (≤6 years vs. > 6 years) using BMI z-scores as the outcome. In the stratified models, all covariates included in the main analysis were adjusted for, except for sex or age, respectively.

Statistical analyses were performed using R software (version 4.5.0). Statistical significance was determined based on a two-tailed *p*-value threshold of < 0.05.

## 3 Results

### 3.1 Population characteristics in this study

The characteristics of study participants are presented in Table 1. The median age of the included pregnant women was 27.5 years. Among the 130 mothers, 66.7% held a bachelor's degree or higher, 78.7% received folic supplementation during pregnancy, 97.5% were primiparous, and 61.4% had a history of passive smoking. The mean age of the included children was 6.49 years, with an average birth weight of 3,423 g. Of the 130 mother-child pairs, 44 children (33.9%) were classified as OWO, with 19 (14.6%) were diagnosed as obese. Mothers of OWO children exhibit significantly higher BMI values compared to those with children of normal weight (*p* = 0.001). Additionally, a higher proportion of boys were observed in the OWO category, and these boys also had higher birth weights. Other demographic characteristics, such as maternal age, parity, outdoor activity and daily consumption of sugary drinks, were comparable between the two groups. The maternal and infant characteristics included in the analysis and those excluded from the analysis are comparable (Table S1).

**Table 1:** Characteristics of study population.

**Characteristic**	**Overall (*n* = 130)**	**Normal (*n* = 86)**	**OWO (*n* = 44)**	***p*-value**
**Mothers**				0.194
Maternal age (years)	27.5 (2.7)	27.7 (2.6)	27.1 (2.8)	
BMI (kg/m^2^)	22.8 (3.3)	22.1 (2.8)	24.2 (3.6)	0.001
< 18.5	11 (8.8%)	10 (12.2%)	1 (2.4%)	0.014
18.5–23.9	72 (57.6%)	51 (62.2%)	21 (48.8%)	
≥24	42 (33.6%)	21 (25.6%)	21 (48.8%)	
**Maternal education**				0.423
High school graduate or lower	43 (33.3%)	25 (29.4%)	18 (40.9%)	
College graduate or higher	86 (66.7%)	60 (70.6%)	26 (59.1%)	
**Personal income**				0.129
< ¥20,000	17 (32.1%)	9 (24.3%)	8 (50.0%)	
≥¥20,000	36 (67.9%)	28 (75.7%)	8 (50.0%)	
**Smoking history**				1.000
No	128 (98.5%)	85 (98.8%)	43 (97.7%)	
Yes	2 (1.5%)	1 (1.2%)	1 (2.3%)	
**Folate supplementation**				0.857
No	27 (21.3%)	19 (22.4%)	8 (19.0%)	
Occasionally	33 (26.0%)	21 (24.7%)	12 (28.6%)	
Frequently	67 (52.7%)	45 (52.8%)	22 (52.4%)	
**Passive smoking history**				0.616
No	78 (61.4%)	54 (63.5%)	24 (57.1%)	
Yes	49 (38.6%)	31 (36.5%)	18 (42.9%)	
**Drinking history**				1.000
No	119 (93.0%)	79 (92.9%)	40 (93.0%)	
Yes	9 (7.0%)	6 (7.1%)	3 (7.0%)	
**Parity**				0.257
0	117 (97.5%)	79 (98.8%)	38 (95.0%)	
≥1	3 (2.5%)	1 (1.2%)	2 (5.0%)	
**Children**				0.491
Age (years)	6.49 (0.95)	6.45 (0.94)	6.57 (0.97)	
**Gender**				0.022
Boys	66 (50.8%)	37 (43.0%)	29 (65.9%)	
Girls	64 (49.2%)	49 (57.0%)	15 (34.1%)	
Birth weight (g)	3,423 (486)	3,348 (471)	3,570 (485)	0.015
Gestational week (weeks)	39.3 (1.4)	39.3 (1.3)	39.3 (1.5)	0.848
BMI *z*-scores	0.51 (1.3)	−0.23 (0.8)	1.98 (0.7)	0.001
**Outdoor activity**				0.810
< 1 h/day	22 (18.6%)	14 (17.9%)	8 (20.0%)	
1–2 h/day	59 (50.0%)	38 (48.7%)	21 (52.5%)	
≥2 h/day	37 (31.4%)	26 (33.3%)	11 (27.5%)	
**Daily sugar-sweetened beverage intake**				0.560
No	61 (51.3%)	43 (53.8%)	18 (46.2%)	
Yes	58 (48.7%)	37 (46.2%)	21 (53.8%)	

**Figure 1:**
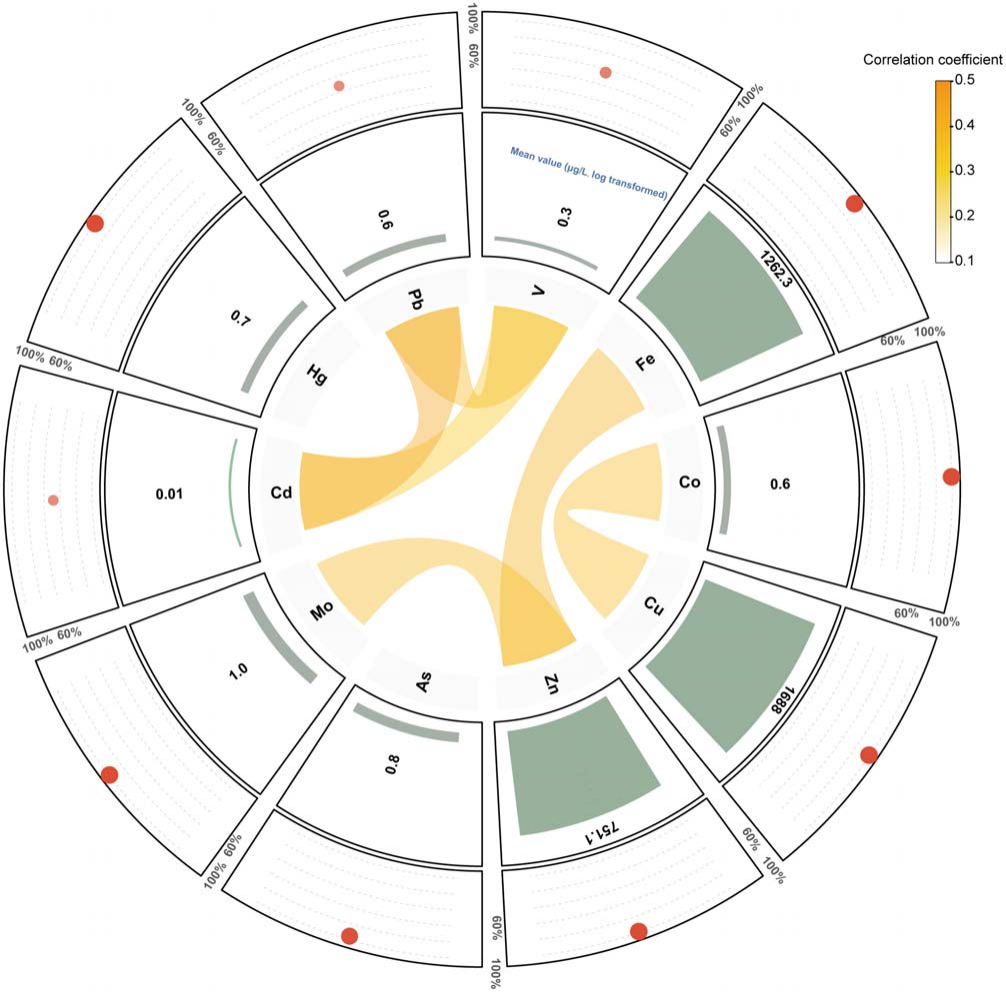
Circular plot illustrating the relationships and exposure profiles of the analyzed metals. The inner ring visualizes the correlation network among metals with correlation coefficients ≥ 0.3 or ≤ -0.3, with the width of the connecting lines proportional to the strength of correlation. The middle ring represents the relative exposure levels of each metal, while the outer ring shows the detection rates for each metal. The size and transparency of the red circles represent the detection rate of each metal, with larger and more opaque circles indicating higher detection frequencies.

Figure 1 illustrates the distribution of serum metal concentrations. Detection rates were generally high (>90%) for most metals, except for vanadium (V, 56.15%), cadmium (Cd, 48.46%), and mercury (Hg, 47.96%). Among the analyzed elements, copper (Cu) demonstrated the highest mean concentration (1,771.14 μg/L), followed by iron (Fe, 1,539.13 μg/L), while cadmium (Cd) had the lowest (0.09 μg/L; Table S2). Pairwise correlation analysis revealed predominantly positive correlations among metals, with coefficients ranging from −0.20 to 0.44.

### 3.2 Associations of individual metal exposure during pregnancy and offspring's OWO risk and BMI z-scores

**Figure 2:**
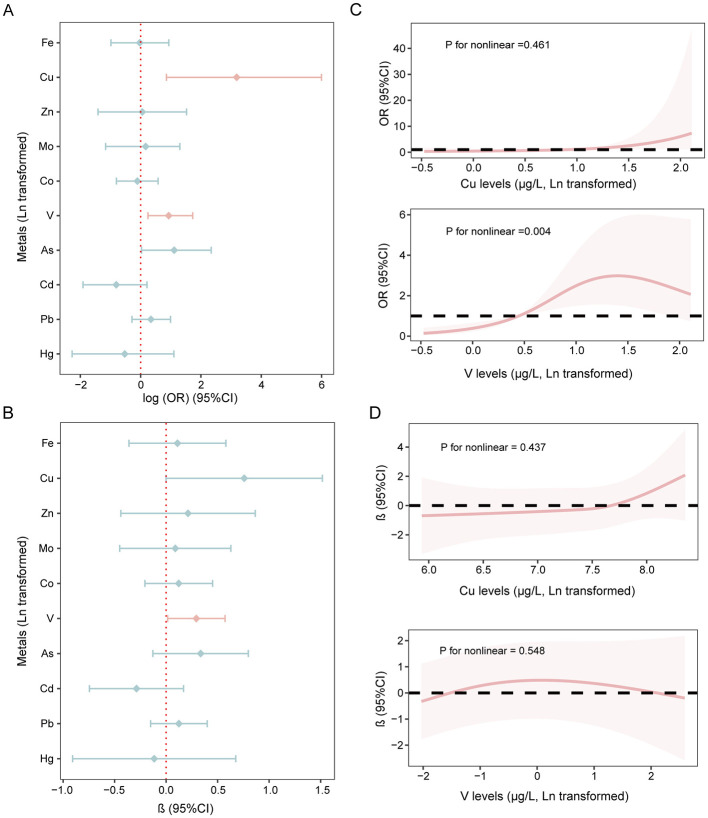
Associations of metals exposure during pregnancy and children's OWO risk BMI *z*-scores value. **(A)** Association between prenatal metals exposure and OWO risk. **(B)** Associations of prenatal metals exposure and BMI *z*-scores of children. **(C)** Dose-response relationship between key metals and OWO risk. **(D)** Dose-response relationship between key metals and BMI *z*-scores. Models were adjusted for maternal age, the pre-pregnancy BMI, maternal education, parity, passive smoking history during pregnancy, children's gender, birth weight, duration of outdoor activity, and the frequency of sugar-sweetened beverage intake.

Logistic regression models revealed significant associations between prenatal exposure to multiple metals and the risk of OWO in children (Figure 2A). Specifically, after adjusting for potential covariates, prenatal Cu and V exposure were significantly associated with higher odds of OWO in school-aged children (Cu: OR = 24.171, 95% *CI*: 2.351–403.256, *p* = 0.014; V: OR = 2.534, 95% *CI*: 1.273–5.623, *p* = 0.014). Additionally, prenatal V exposure showed a positive association with BMI *z*-scores in this population (β: 0.293, 95% *CI*: 0.015–0.572, *p* = 0.042), while prenatal Cu exposure showed a borderline positive association (β: 0.758, 95% *CI*: −0.001–1.517, *p* = 0.054; Figure 2B). Sex-stratified analyses indicated that maternal V exposure was positively associated with BMI *z*-scores in boys, whereas maternal Cu exposure was positively associated with BMI *z*-scores in girls (Figure S1). Further stratification by children's age revealed that maternal V and Cu exposure were positively associated with BMI *z*-scores in children older than 6 years (Figure S2), whereas no associations were observed in children younger than 6 years.

RCS model indicated a non-linear relationship between V levels and OWO risk (Figure 2C, *p* for nonlinear = 0.004), wherein low concentrations of V were associated with an increased risk of OWO, while higher concentrations appeared to exert a protective effect. No other significant non-linear associations were identified between either two metals and children's OWO or BMI *z*-scores (Figures 2C, D).

### 3.3 Mixture analysis of metals and child OWO risk and BMI z-scores

**Figure 3:**
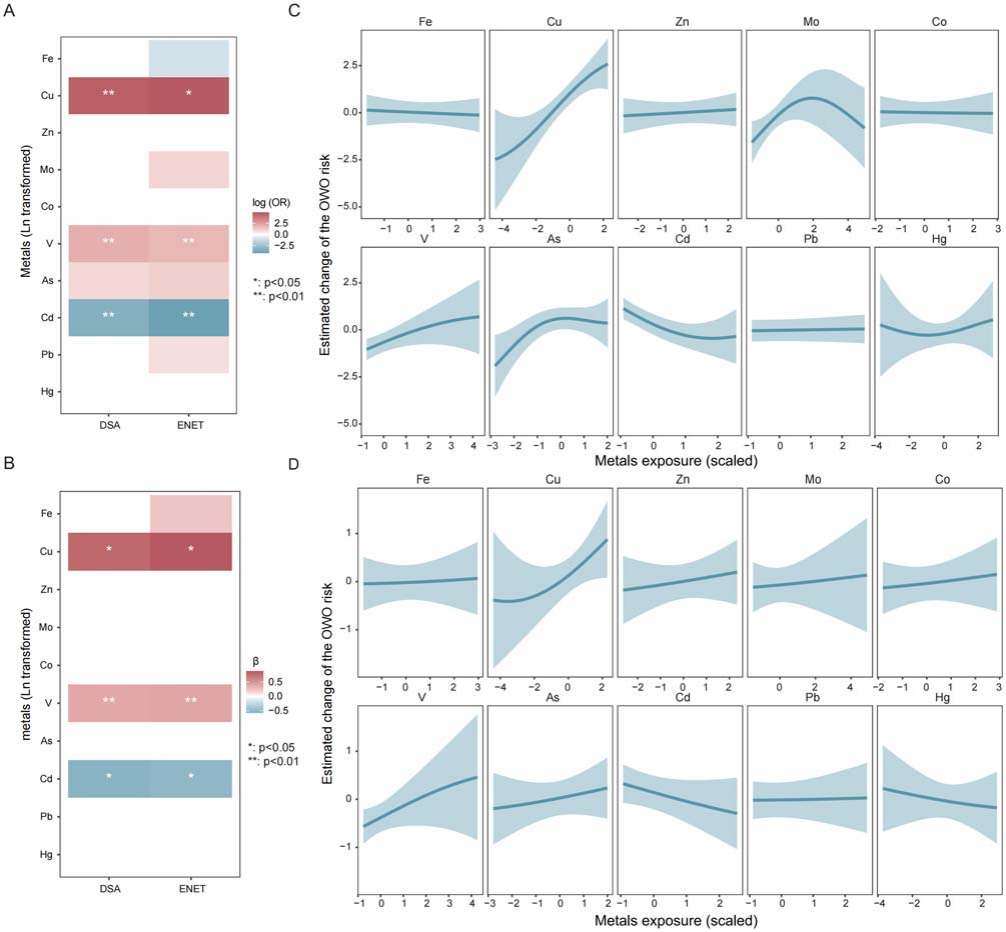
Associations of metal mixture and BMI *z*-scores or OWO risk. **(A)** Results of the DSA and ENET model for the association between metal mixed exposure and OWO risk. **(B)** Results of the DSA and ENET model for the association between metal mixed exposure and BMI *z*-scores. **(C)** Univariate exposure–response functions (estimates and 95% CI) between exposure to single metal and OWO when other metals are fixed at the 50th percentile. **(D)** Univariate exposure–response functions (estimates and 95% CI) between exposure to single metal and BMI *z*-scores when other metals are fixed at the 50th percentile. Models were adjusted for maternal age, the pre-pregnancy BMI, maternal education, parity, passive smoking history during pregnancy, children's gender, birth weight, duration of outdoor activity, and the frequency of sugar-sweetened beverage intake.

Variable selection method identified Cu and V as being positively associated an increased risk of childhood OWO, whereas Cd exposure demonstrated a negative association with OWO risk (Figure 3A). A comparable trend was observed for BMI *z*-scores (Figure 3B), whereby higher BMI *z*-scores were noted when all metals were above the 50th percentile relative to their median exposure levels (Figure S3). With respect to PIP values, Cu, As, and Hg exhibit the highest conPIP values (conPIP > 0.8) for their association with OWO. Among metals with groupPIP > 0.5 for BMI *z*-scores, Cu demonstrated the highest conPIP values (0.79, Table S3), underscoring its prominent role in influencing child growth. Furthermore, univariate exposure-response analysis revealed approximately linear associations of key metals with both OWO and BMI *z*-scores. No interactive effect between metals on OWO risk was identified in bivariate exposure response analysis (Figures S4, S5). Specifically, Cu and V were found to be positively associated with OWO risk (Figure 3C), and their concentrations were likewise positively correlated with elevated BMI *z*-scores in school-aged children (Figure 3D).

### 3.4 Metabolome-wide association study on maternal serum metals and children's OWO and BMI z-scores

**Figure 4:**
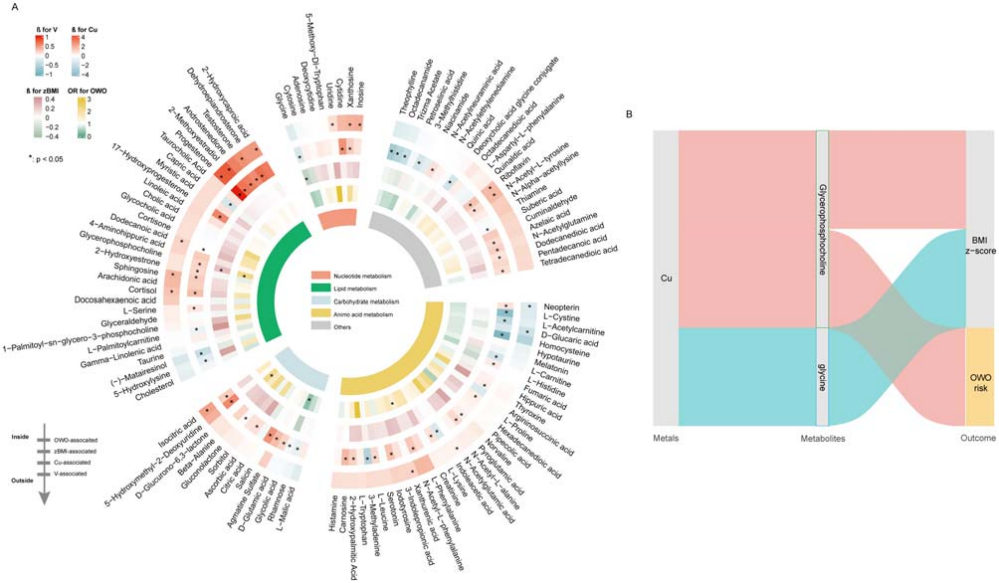
Association of key metals, metabolites and OWO risk and BMI *z*-scores. **(A)** Heatmaps depicting the associations of metabolites with V, Cu and BMI *z*-scores. **(B)** Sankey diagram illustrating key metabolites involved in the associations between Cu exposure and OWO risk as well as BMI *z*-scores.

Of the 170 metabolites detected, prenatal Cu and V were significantly associated with 79 and 17 metabolites, respectively (*p* < 0.05, Figure 4A). Higher levels of Cu were primarily associated with lipid metabolism. Notable associations included positive correlations with glycerophosphocholine, 17-hydroxyprogesterone, and 2-hydroxycaproic acid, alongside negative associations with 5-hydroxylysine, capric acid, and L-serine. Similarly, V exposure was predominantly associated with metabolites involved in lipid metabolic processes, such as 2-hydroxycaproic acid, 2-methoxyestradiol, cortisol, dodecanoic acid, sphingosine, progesterone, and testosterone.

In the Metabolite-Wide Association Study (MWAS) of childhood OWO and BMI *z*-scores, elevated levels of D-Glucurono-6,3-lactone, glycerophosphocholine, glycocholic acid, and L-Phenylalanine were significantly associated with an increased risk of OWO. In addition, glycerophosphocholine was positively associated with children's BMI *z*-scores, whereas glycine exhibited a negative association (Figure 4A).

Based on the MITM approach, glycerophosphocholine was identified in the MWAS of maternal Cu exposure, as well as in the MWAS for both children's OWO risk and BMI *z*-scores (Figure 4B). Moreover, glycine showed a significant association with maternal Cu levels and a negative association with children's BMI *z*-scores, indicating its potential mediating role in the relationship between prenatal Cu exposure and children's growth outcomes.

## 4 Discussion

Based on a prospective birth cohort study, we performed a metabolomics analysis of maternal serum to investigate potential metabolites involved in the association between prenatal metal exposure and the risk of OWO in children at school age. Our findings indicated that prenatal metal mixtures exposure was positively associated with both the OWO risk and BMI *z*-scores in children, with Cu and V identified as key contributing elements. Metabolites such as glycerophosphocholine and glycine played potential intermediate roles in the association between maternal Cu levels and OWO risk and BMI *z*-scores in children. These findings underscored the potential mechanistic role of maternal metabolites in the association between prenatal exposure to metal mixtures and childhood adiposity outcomes.

In comparison with global data on maternal serum metal concentrations, the levels observed in this study were broadly comparable to internal exposure levels documented among pregnant populations in China (33), Brazil (34), Norway (35) and the United States (36). However, serum concentrations of As and Cd in our cohort were lower than those documented in studies from China (37–39). These discrepancies may be attributable to population heterogeneity, highlighting the need for a comprehensive, cross-national investigation to better characterize global patterns of prenatal metal exposure.

We observed that maternal Cu exposure was associated with an increased risk of OWO in childhood, consistent with previous results reported in the U.S. populations (15, 40). The exposure–response curve for Cu in this study suggested a threshold-like pattern, with little association at lower exposure levels and increased risk at higher concentrations. As an essential trace element and nutrient during pregnancy, Cu plays a critical role in several cellular pathways including redox reactions and energy production (41). Adequate Cu levels are necessary for maintaining metabolic homeostasis, which may partly explain the initially flat association (42). Dysregulation of Cu homeostasis has been associated with metabolic abnormalities, such as glucose intolerance and hyperlipidemia (43). Such metabolic perturbations during pregnancy have been linked to an elevated risk of childhood obesity (44, 45). Recent epidemiological studies have further reported non-linear associations between Cu exposure and obesity-related outcomes, supporting the biological plausibility of this pattern (46). These findings highlight the importance of considering potential exposure thresholds when evaluating metabolic effects of trace metals. Maternal Cu exposure has been implicated in DNA methylation at CpGs sites within the Robust Placental Clock, thereby modulating the expression of genes related to energy balance and placental inflammatory pathways (47). These epigenetic modifications may subsequently affect fetal trajectories and body composition in the offspring (48). Our findings revealed that glycerophosphocholine and glycine may mediate the relationship between maternal Cu levels and OWO risk as well as BMI *z*-scores in children. Glycerophosphocholine, a natural choline compound commonly found in dietary supplements, has been shown in MWAS to correlate with metal exposure (49). Elevated glycerophosphocholine levels have been related to increased growth hormone secretion and hepatic fat oxidation (50), as well as to several cardiovascular disease risk factors (51). Maternal choline supplementation has been reported to modulates placental nutrient transporter and nutrient metabolism, thereby affecting nutrient supply to the developing fetus (52). Glycine, a dispensable amino acid during pregnancy (53), was also found to be a potential mediator in the association between maternal Cu exposure and BMI *z*-scores in our study. Lower maternal glycine levels have been associated with the pathogenesis of metabolic disorders, including obesity and diabetes (54), both of which are established risk factors for childhood obesity (55, 56). Collectively, these findings highlighted the potential role of maternal choline and glycine metabolism in shaping childhood growth trajectories.

Our results indicated that prenatal exposure to V was positively correlated with higher BMI *z*-scores in children. V is naturally occurring trace mineral widely utilized in industrial applications due to its distinctive physical and chemical properties (57). We found a non-linear association between prenatal V exposure and offspring OWO, characterized by an inverted U-shaped pattern. V has been reported to exert insulin-mimetic effects at lower doses but impair metabolic homeostasis at higher levels, providing biological plausibility for the observed pattern (58). Similar non-linear associations between prenatal V exposure and developmental outcomes have also been reported in recent epidemiological studies (59), and restricted fetal growth is a recognized risk factor for obesity throughout the life course (60). Findings from a prospective cohort study further suggested that prenatal V exposure may exert negative effects on early postnatal growth (61). Our findings underscore the need for non-linear modeling approaches in studies of prenatal metal exposure and childhood obesity. The biological mechanism through which excessive V exposure during pregnancy influences child growth remains unclear. However, V present in maternal serum can cross the placental barrier and accumulate in the fetal environment (62). A population-based study identified associations between V exposure and biomarkers of inflammation and oxidative stress (63), both of which are implicated in the development of obesity (64). Further studies are warranted to clarify the biological mechanisms underlying the potential effects of V exposure on fetal development and postnatal growth.

Sex- and age-stratified analyses suggested potential heterogeneity in the associations between maternal metal exposure and child growth. Sex-specific patterns are biologically plausible, as fetal growth and postnatal metabolic regulation differ by sex due to variations in endocrine function, body composition development, and metal toxicokinetic (65). Sex-specific associations between prenatal metal exposure and child growth have been reported previously (66). More studies with large sample size are required to confirm our results. Moreover, positive associations of maternal V and Cu exposure with BMI z-scores were observed primarily among children aged ≥6 years, suggesting that prenatal programming effects may become more apparent later in childhood. This pattern is consistent with the developmental origins of health and disease (DOHaD) framework, which posits that early-life exposures can have long-term metabolic consequences (67). These findings underscore the importance of considering sex and age as potential effect modifiers when evaluating the growth effects of prenatal metal exposure.

This study comprehensively examined the mediating role of metabolites linking prenatal metal mixtures to childhood OWO and BMI *z-*scores. Several methodological strengths enhance the validity of our findings. Notably, we employed an integrative analytical framework combining single-exposure, variable selection, and mixture modeling approaches to assess the relationships between metal exposures and child anthropometric outcomes, reducing potential biases inherent to individual modeling strategies. Second, the integration of metabolomic data provided mechanistic insights and offers a foundation for future research into the biological pathways linking prenatal exposures and child health outcomes. Despite the strengths, this study has certain limitations. The assessment of maternal metal exposure relied on a single serum sample obtained during pregnancy, which may not fully reflect temporal variability. Future research should include serial measurements across different gestational periods to better capture dynamic exposure profiles and critical developmental windows. Additionally, although we adjusted for several prenatal and postnatal confounders, data on postnatal dietary intake, including fat consumption, were not collected. Larger-scale studies across diverse populations studies are warranted to confirm the generalizability of our findings.

## 5 Conclusion

In summary, this study provided evidence that prenatal exposure to metal mixtures, particularly Cu and V, contributed to an increased risk of childhood OWO and elevated BMI *z*-scores. Through integrative metabolomics analysis, we identified glycerophosphocholine and glycine as potential mediators linking maternal metal exposure to adverse childhood growth outcomes. These findings highlight the biological plausibility of metabolic disruptions during pregnancy contributing to long-term health risks in offspring. Future research involving repeated exposure measurements and diverse populations is essential to validate these findings and better understand the underlying mechanisms of metal-related developmental programming.

## Data Availability

Data will be made available on request. Requests to access these datasets should be directed to yyy2001@njmu.edu.cn.
