# Supplementary material for: Prenatal exposure to metal mixtures and the risk of overweight and obesity in school-aged children: insights from metabolomic profiling
Source: Front Nutr. 2026 Feb 24;13:1762885. doi: 10.3389/fnut.2026.1762885 (PMC12971668; doi:10.3389/fnut.2026.1762885)
Supplement: Supplementary file 1 [file Table_1.docx]

**Supplementary materials to**

**Prenatal Exposure to Metal Mixtures and the Risk of Overweight and Obesity in School-Aged Children: Insights from Metabolomic Profiling**

Xu Yang ^1, #^, Xian Sun ^4, #^, Lili Bao ^1^, Yuwen Lv ^1^, Yanyan Yu^1, *^, Yankai Xia ^2, 3, *^

^1^Suzhou Affiliated Hospital of Nanjing Medical University, Suzhou Municipal Hospital, Gusu School, Nanjing Medical University, Suzhou, China

^2^State Key Laboratory of Reproductive Medicine and Offspring Health, School of Public Health, Nanjing Medical University, Nanjing, China

^3^Key Laboratory of Modern Toxicology of Ministry of Education, School of Public Health, Nanjing Medical University, Nanjing, China

^4^Ligang Hospital of Jiangyin City, Wuxi, China

^#^: These authors contributed equally to this work.

^*^ Corresponding authors at:

State Key Laboratory of Reproductive Medicine and Offspring Health, School of Public Health, Nanjing Medical University, No.101 Longmian Road, Nanjing, 211166, China. E-mail addresses: yankaixia@njmu.edu.cn (Y. Xia)

Suzhou Affiliated Hospital of Nanjing Medical University, Suzhou Municipal Hospital, Gusu School, Suzhou, 215031, China. E-mail addresses: yyy2001@njmu.edu.cn (Y.Yu)

Table S1. Comparison of characteristics between included and excluded participants

| Characteristic | Included (n=130) | Not included (n=514) | P-value |
| --- | --- | --- | --- |
| ***Mothers*** |  |  |  |
| Maternal age (years) | 27.5 (2.68) | 27.6 (2.96) | 0.812 |
| BMI (kg/m2) | 22.8 (3.28) | 23.0 (3.15) | 0.534 |
| <18.5 | 11 (8.8%) | 26 (5.1%) | 0.294 |
| 18.5-23.9 | 72 (57.6%) | 300 (59.3%) |  |
| ≥24 | 42 (33.6%) | 180 (35.6%) |  |
| Maternal education |  |  | 0.074 |
| High school graduate or lower | 44 (33.3%) | 140 (27.5%) |  |
| College graduate or higher | 86 (66.7%) | 369 (72.5%) |  |
| Personal income |  |  | 0.179 |
| <20,000 | 17 (32.1%) | 54 (22.2%) |  |
| ≥20,000 | 36 (67.9%) | 189 (77.8%) |  |
| Smoking history |  |  | 1.000 |
| No | 128 (98.5%) | 500 (98.0%) |  |
| Yes | 2 (1.5%) | 10 (2.0%) |  |
| Folate supplementation |  |  | 0.328 |
| No | 27 (21.3%) | 140 (27.5%) |  |
| Occasionally | 33 (26.0%) | 130 (25.5%) |  |
| Frequently | 67 (52.8%) | 239 (47.0%) |  |
| Passive smoking history |  |  | 0.380 |
| No | 78 (61.4%) | 331 (66.1%) |  |
| Yes | 49 (38.6%) | 170 (33.9%) |  |
| Drinking history |  |  | 1.000 |
| No | 119 (93.0%) | 471 (92.9%) |  |
| Yes | 9 (7.0%) | 36 (7.1%) |  |
| Parity |  |  | 0.588 |
| 0 | 117 (97.5%) | 384 (95.8%) |  |
| ≥1 | 3 (2.5%) | 17 (4.2%) |  |
| ***Children*** |  |  |  |
| Age (years) | 6.49 (0.95) | 6.55 (0.88) | 0.661 |
| Gender |  |  | 0.708 |
| Boys | 66 (50.8%) | 193 (48.4%) |  |
| Girls | 64 (49.2%) | 206 (51.6%) |  |
| Birth weight (g) | 3423 (486) | 3372 (438) | 0.287 |
| Gestational week (weeks) | 39.3 (1.35) | 39.1 (1.77) | 0.188 |
| BMI (kg/m2) | 16.5 (2.35) | 16.2 (3.11) | 0.523 |
| Outdoor activity |  |  | 0.429 |
| <1 hour/day | 22 (18.6%) | 14 (26.9%) |  |
| 1-2 hours/day | 59 (50.0%) | 25 (48.1%) |  |
| ≥2 hours/day | 37 (31.4%) | 13 (25.0%) |  |
| Daily consumption of sugary drinks |  |  | 0.418 |
| No | 61 (51.3%) | 24 (49.0%) |  |
| Yes | 58 (48.7%) | 25 (51.0%) |  |

Table S2. Concentrations of trace elements in serum.

| Metals (μg/L) | LOD | Detection rate (%) | Mean (SD) | Median (IQR) |
| --- | --- | --- | --- | --- |
| V | 0.26 | 56.15 | 0.53 (1.33) | 0.29 (0.33) |
| Fe | 43.51 | 100.00 | 1539.13 (1078.66) | 1262.29 (870.85) |
| Co | 0.07 | 100.00 | 0.98 (0.95) | 0.58 (0.8) |
| Cu | 5.97 | 100.00 | 1771.41 (551.8) | 1688 (682.97) |
| Zn | 86.38 | 100.00 | 844.64 (357.79) | 751.07 (459.38) |
| As | 0.31 | 95.38 | 1 (0.64) | 0.85 (0.67) |
| Mo | 0.18 | 100.00 | 1.34 (1.59) | 1.04 (0.49) |
| Cd | 0.08 | 48.46 | 0.09 (0.06) | 0.04 (0.08) |
| Hg | 0.09 | 100.00 | 0.66 (0.19) | 0.67 (0.17) |
| Pb | 1.25 | 47.69 | 2.72 (5.47) | 0.62 (2.07) |

Abbreviations: LOD, the limits of detection; V, vanadium; Fe, iron; Co, cobalt; Cu, copper; Zn, zinc; As, arsenic; Mo molybdenum; Cd, cadmium; Hg, mercury; Pb, lead

Table S3. PIP values of metals in BKMR models

| Metals | group | Overweight | |  | BMI z-score | |
| --- | --- | --- | --- | --- | --- | --- |
|  |  | groupPIP | condPIP |  | groupPIP | condPIP |
| Fe | 1 | 0.82 | 0.08 |  | 0.28 | 0.29 |
| Cu | 2 | 0.94 | 0.88 |  | 0.55 | 0.79 |
| Zn | 1 | 0.82 | 0.13 |  | 0.28 | 0.43 |
| Mo | 1 | 0.82 | 0.79 |  | 0.28 | 0.28 |
| Co | 2 | 0.94 | 0.12 |  | 0.55 | 0.21 |
| V | 3 | 0.96 | 0.26 |  | 0.38 | 0.49 |
| As | 4 | 0.84 | 1.00 |  | 0.22 | 1.00 |
| Cd | 3 | 0.96 | 0.73 |  | 0.38 | 0.35 |
| Pb | 3 | 0.96 | 0.02 |  | 0.38 | 0.16 |
| Hg | 5 | 0.93 | 1.00 |  | 0.15 | 1.00 |

Abbreviations: V, vanadium; Fe, iron; Co, cobalt; Cu, copper; Zn, zinc; As, arsenic; Mo molybdenum; Cd, cadmium; Hg, mercury; Pb, lead


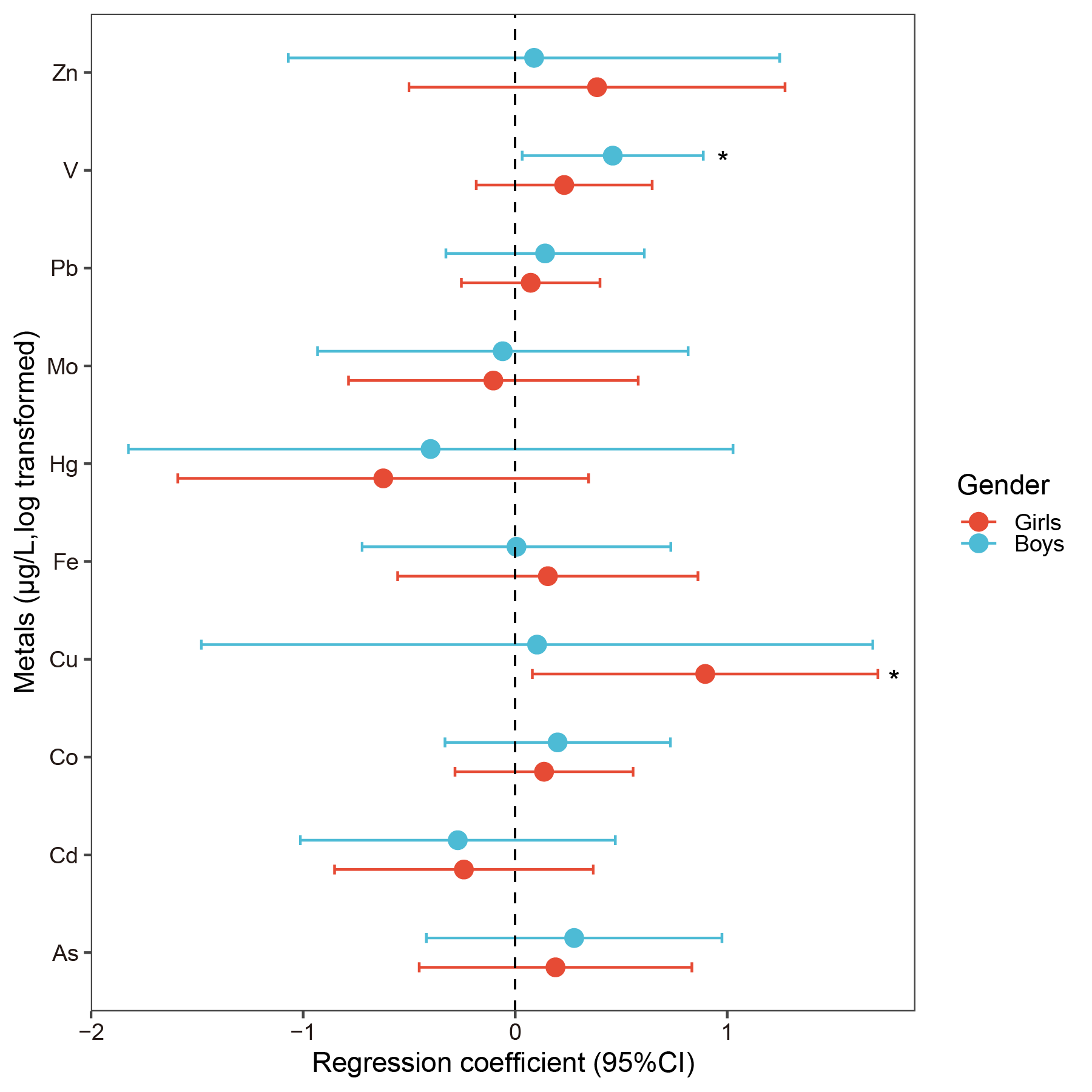


Figure S1. Association between maternal metal exposure and BMI *z*-score stratified by children’s gender.


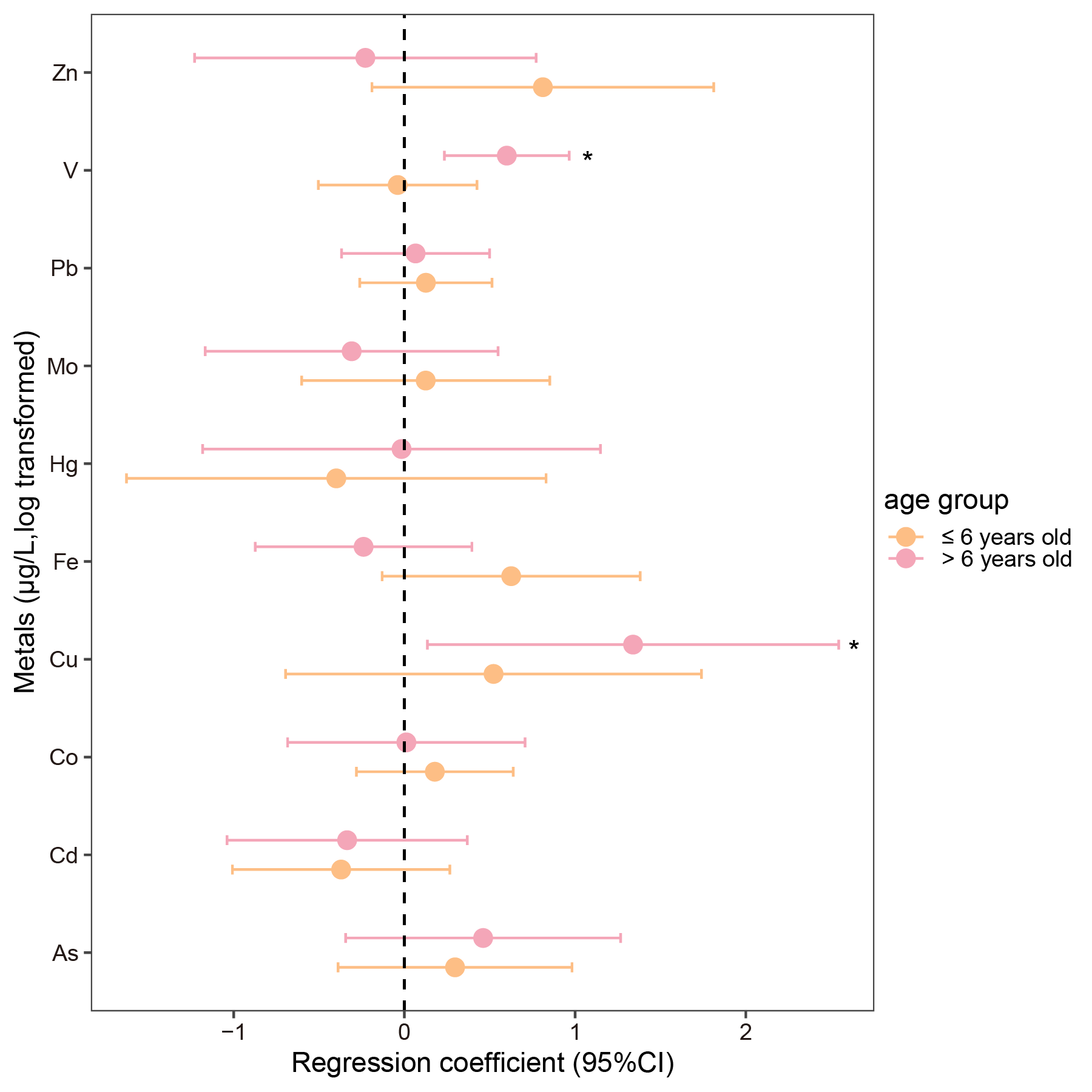


Figure S2. Association between maternal metal exposure and BMI *z*-score stratified by age.


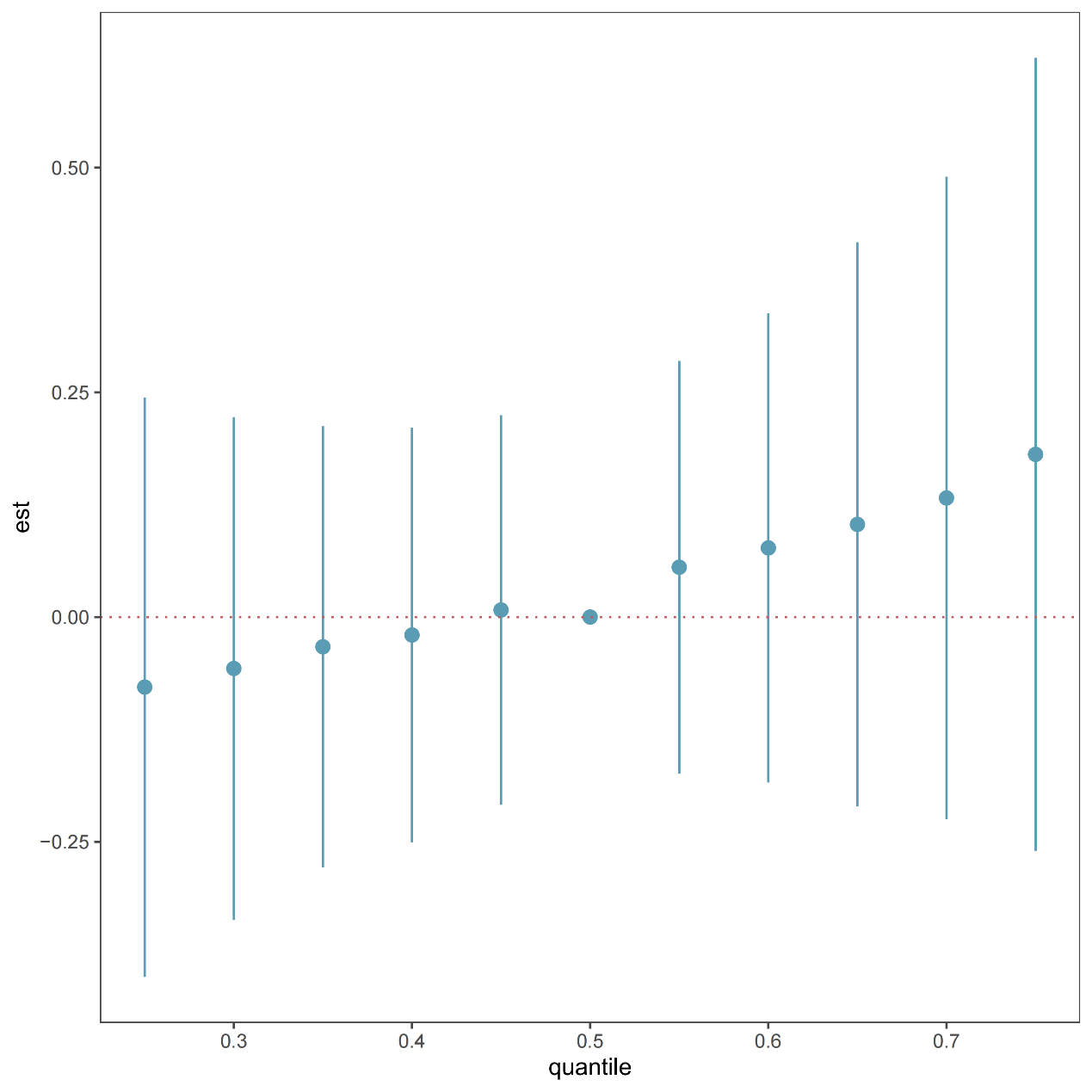


Figure S3. Association of metal mixture and BMI *z*-scores using BKMR model.


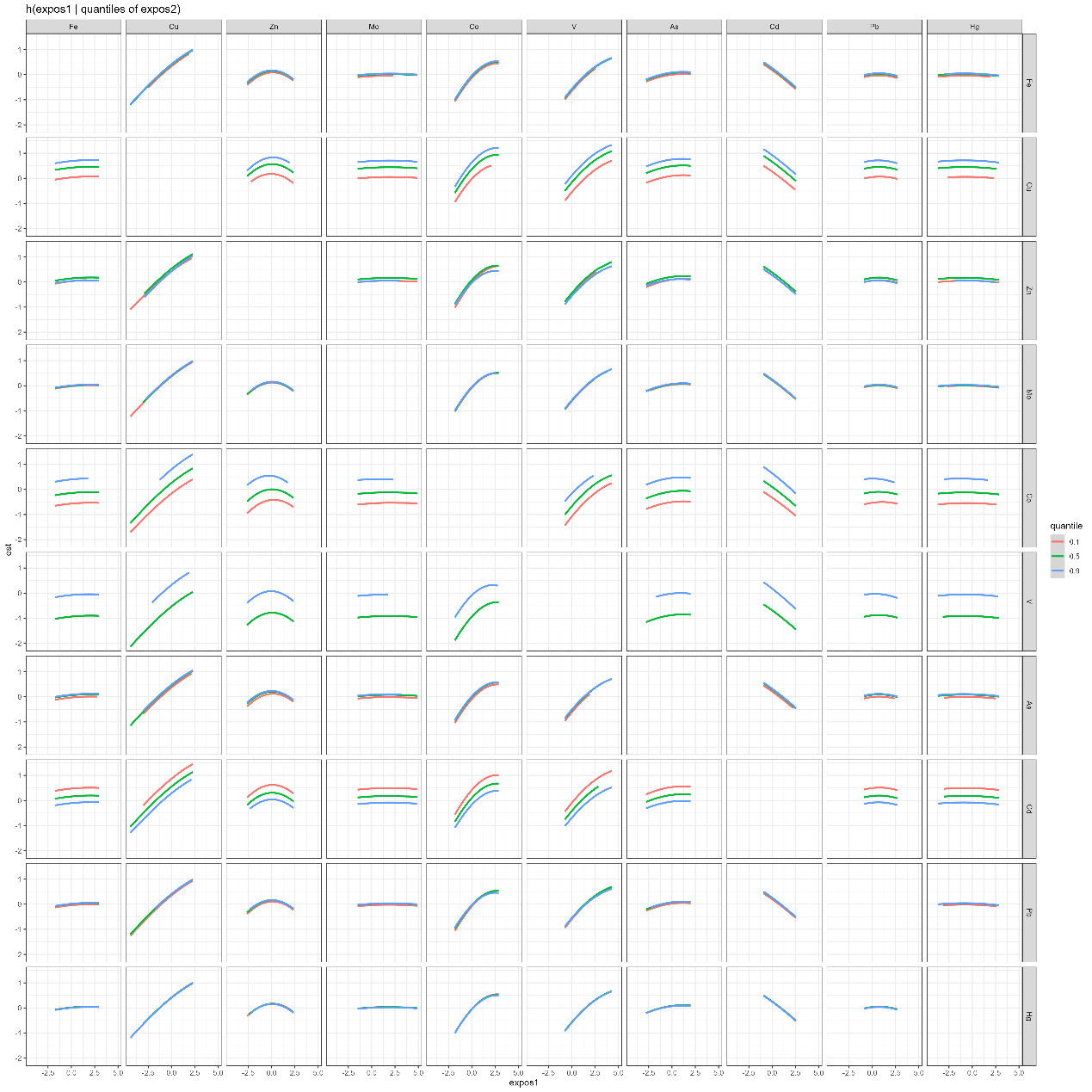


Figure S4. BKMR analysis of interactive effects of metal exposure on OWO risk. Bivariate exposure response functions of every two elements in OWO risk. Bivariate exposure–response functions for: exposure 1 when exposure 2 is fixed at either the 10th, 50th, or 90th percentiles and other trace elements are fixed at the 50th percentile. Models were adjusted for maternal age, the pre-pregnancy BMI, maternal education, parity, passive smoking history during pregnancy, children’s gender, birth weight, duration of outdoor activity, and the frequency of sugar-sweetened beverage intake.


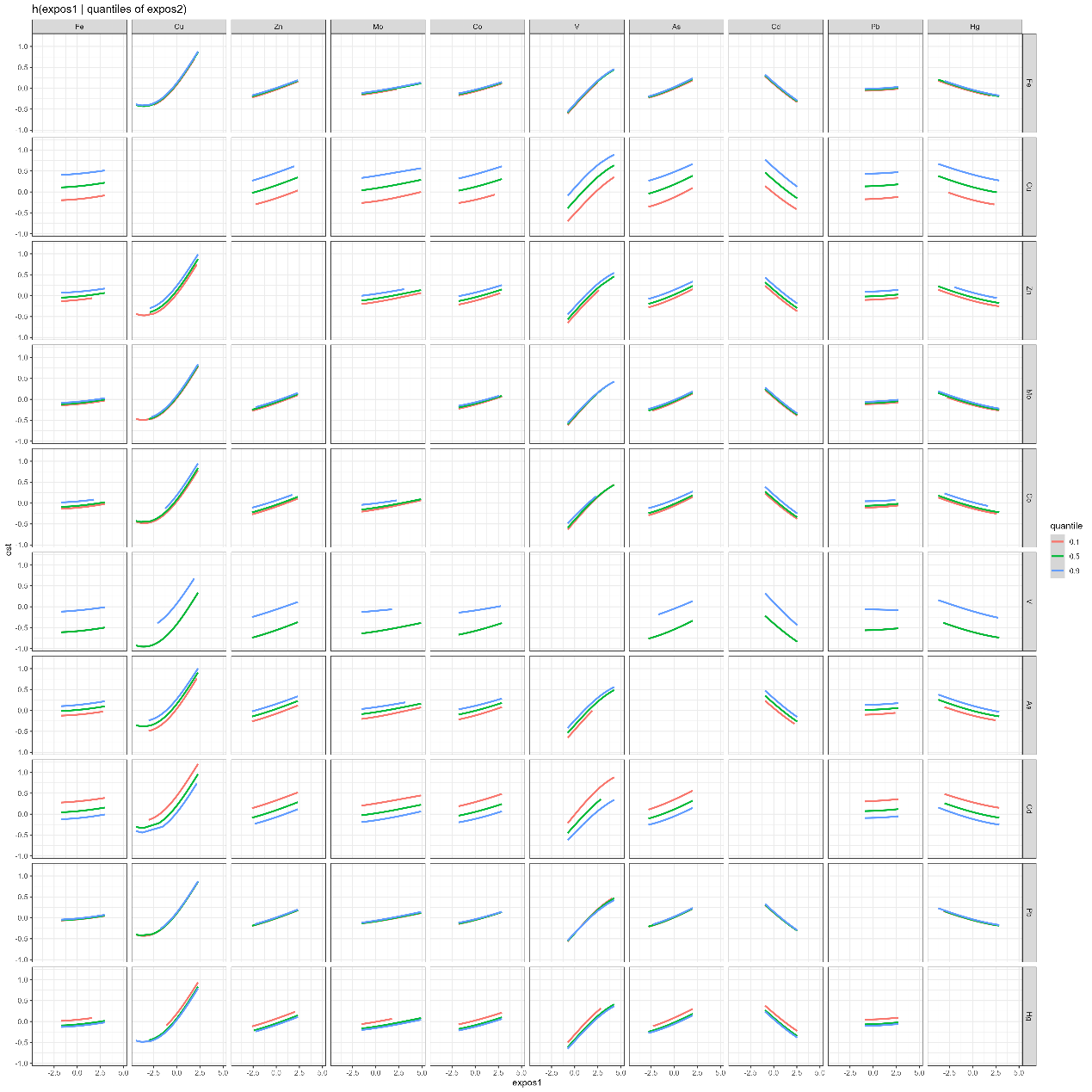


Figure S5. BKMR analysis of interactive effects of metal exposure on BMI z-scores. Bivariate exposure response functions of every two elements in OWO risk. Bivariate exposure–response functions for: exposure 1 when exposure 2 is fixed at either the 10th, 50th, or 90th percentiles and other trace elements are fixed at the 50th percentile. Models were adjusted for maternal age, the pre-pregnancy BMI, maternal education, parity, passive smoking history during pregnancy, children’s gender, birth weight, duration of outdoor activity, and the frequency of sugar-sweetened beverage intake.
